# Protecting HIV information in countries scaling up HIV services: a baseline study

**DOI:** 10.1186/1758-2652-14-6

**Published:** 2011-02-06

**Authors:** Eduard J Beck, Sundhiya Mandalia, Guy Harling, Xenophon M Santas, Debra Mosure, Paul R Delay

**Affiliations:** 1 Programme Branch, UNAIDS, Geneva, Switzerland; 2 HIV/GUM Department, Imperial College London, UK; 3 Division of Global HIV/AIDS, Centers for Disease Control and Prevention, Atlanta, Georgia, USA

## Abstract

**Background:**

Individual-level data are needed to optimize clinical care and monitor and evaluate HIV services. Confidentiality and security of such data must be safeguarded to avoid stigmatization and discrimination of people living with HIV. We set out to assess the extent that countries scaling up HIV services have developed and implemented guidelines to protect the confidentiality and security of HIV information.

**Methods:**

Questionnaires were sent to UNAIDS field staff in 98 middle- and lower-income countries, some reportedly with guidelines (G-countries) and others intending to develop them (NG-countries). Responses were scored, aggregated and weighted to produce standard scores for six categories: *information governance, country policies*, *data collection*, *data storage, data transfer *and *data access*. Responses were analyzed using regression analyses for associations with national HIV prevalence, gross national income per capita, OECD income, receiving US PEPFAR funding, and being a G- or NG-country. Differences between G- and NG-countries were investigated using non-parametric methods.

**Results:**

Higher *information governance *scores were observed for G-countries compared with NG-countries; no differences were observed between *country policies *or *data collection *categories. However, for *data storage, data transfer *and *data access*, G-countries had lower scores compared with NG-countries. No significant associations were observed between country score and HIV prevalence, per capita gross national income, OECD economic category, and whether countries had received PEPFAR funding.

**Conclusions:**

Few countries, including G-countries, had developed comprehensive guidelines on protecting the confidentiality and security of HIV information. Countries must develop their own guidelines, using established frameworks to guide their efforts, and may require assistance in adapting, adopting and implementing them.

## Background

Many middle- and lower-income countries are scaling up HIV prevention, treatment, care and support services within the context of Universal Access [1] and achieving the Millennium Development Goals [2]. This involves collecting individual-level data, which enable individuals to be tracked over time within and between sites for clinical management, and can also provide information for monitoring or evaluating services. Paper-based and electronic information systems, increasingly developed and used in these countries, must ensure the confidentiality and security of these data, yet allow relatively easy access to such data for both service provision and monitoring and evaluation.

Confidentiality and security must be ensured for data collection, storage, use and dissemination within countries and at international levels. This includes the physical protection of data to guard against environmental threats, such as floods, fire or other environmental threats, and the protection needed to guard against inappropriate use by humans of sensitive information, whether due to inadvertent or deliberate activities.

To improve health outcomes and reduce harm, individual health data must be used to inform healthcare. This involves an ongoing process of refining the balance between:

a) Maximizing benefits that can and should come from the wise and fullest use of data

b) Minimizing the harm that can result from either malicious or inadvertent inappropriate release of individually identifiable data.

These potential benefits and harms may accrue to individuals, groups or institutions. While longitudinal paper-based or electronic patient health data repositories can provide the basic information to monitor and evaluate service provision, they also provide opportunities for breaches in confidentiality of individual records in consolidated and centrally accessible data. These considerations motivated the development of a set of principles or guidelines, which, independent of context, may help maintain the balance between maximizing benefit and minimizing harm. To assist countries in addressing this critical issue, a consensus workshop was held in Geneva, Switzerland, in May 2006, attended by national and international experts, which resulted in the development and publication of the Joint United Nations Programme on HIV/AIDS (UNAIDS) and the US President's Emergency Plan for AIDS Relief (PEPFAR) Interim Guidelines on Protecting the Confidentiality and Security of HIV Information [3].

The issues described and the solutions proposed by the Interim Guidelines go well beyond HIV-information systems [4], and they were developed with the intention that the guidelines would also be relevant for health sector-wide information systems [5].

The Interim Guidelines focus on the three interrelated concepts of privacy, confidentiality and security, all of which affect the protection of sensitive data. Privacy is both a legal and an ethical concept. The legal concept refers to the legal protection that has been accorded to an individual, based on human rights principles [3], to control both access to and use of personal information; it provides the overall framework within which both confidentiality and security are implemented. Confidentiality relates to the right of individuals to have their data protected during collection, storage, transfer and use in order to prevent unauthorized disclosure of that information to third parties. Security refers to a collection of technical approaches that address issues covering physical, electronic and procedural aspects of protecting information collected as part of the scale up of HIV services. Security must support protection of data from both inadvertent and malicious inappropriate disclosure, and minimize data outages due to system failure and user errors.

The focus of this study was, therefore, to assess how middle- and lower-income countries have so far dealt with securing the confidentiality of HIV information through the development of privacy laws, which cover the different types of data collected through their HIV clinical care monitoring and evaluation systems, and the specific security measures identified within the data collection, storage, transfer and analysis process.

## Methods

In September 2007, questionnaires were sent out to field staff present in all 80 countries with UNAIDS offices, which covered 98 middle- and lower-income countries as some of the offices covered more than one country. UNAIDS staff, in conjunction with relevant country staff employed through PEPFAR, were asked to identify the most appropriate country professional(s) to complete the questionnaire. Respondents were contacted by the UNAIDS staff, and UNAIDS or PEPFAR staff were asked to facilitate completion of the questionnaire by country professionals. Questionnaires were returned to the respective UNAIDS country staff member, who reviewed the responses and followed up when necessary with the country respondent.

Subsequently, the completed questionnaires were forwarded to the UNAIDS Secretariat in Geneva. In Geneva, questionnaires were reviewed and if queries arose, country staff was again contacted to try to obtain answers to the queries. Questionnaires were initially piloted in four countries, subsequently revised, and English, French, Russian, Spanish and Portuguese language versions produced. The data collection period was from September 2007 to April 2008.

A substantial number of country respondents indicated that their countries had already developed such guidelines, while the majority acknowledged that such guidelines did not yet exist in their countries. For this reason, two questionnaires were developed at the request of the country respondents themselves: one for countries that reportedly had developed relevant guidelines (G-countries); and another one for countries that had not yet developed such guidelines but intended to do so (NG-countries). Whether a G-country or NG-country questionnaire was to be completed was agreed after discussions between the country respondent and their UNAIDS liaison officer. The two questionnaires covered similar topics, with questions for G-countries phrased in terms of "have you included...", whereas questions for NG-countries were phrased in terms of "would you include...".

Each questionnaire covered the following three areas:

1. The existence of privacy laws in the country

2. The extent to which countries have been able to develop and implement a national HIV monitoring and evaluation system as part of the Three Ones principles that promote better coordination of national responses to their HIV epidemic [6]

3. The physical and electronic protection of data, the conditions of the use of data and release of analyses based on these data.

These areas covered the various measures highlighted in the Interim Guidelines that countries can take to scale up services while improving the confidentiality and security of HIV information.

Some of the analyses produced compared responses between G- and NG-countries. The responses from NG-countries can be interpreted as those topics that respondents would "ideally" like to see included in future guidelines and can be characterized as a "vision statement". The responses from G-countries, meanwhile, provided some "reality check" in terms of what policies countries had actually developed and implemented. The null-hypothesis tested in this study was that there were no significant differences in terms of the guidelines that G-countries had developed and those that NG-countries indicated that they would include in the future.

Questions were aggregated into six related categories, each of which dealt with an important measure for securing the confidentiality of HIV information: *information governance, country policies*, *data collection*, *data storage, data transfer *and *data access*. The scoring system assigned "1" to a positive response, while negative or missing responses received a score of "0". Scores were summed and then standardized so that scores for each category ranged from 0 to 100, where a score of 100 indicated positive responses to all items. Standardization allowed the individual category scores to be compared across respondents.

Standardized composite country scores are presented as median and interquartile (IQR) ranges. Associations between country scores and country HIV prevalence [7], gross national income (GNI) per capita [8], Organization for Economic Cooperation and Development (OECD) income classification [9] and funding received from PEPFAR were investigated.

Comparisons were analysed using non-parametric tests, including the Chi-square test with Yates' correction, Mann-Whitney U and Kruskal-Wallis tests. Standardized composite scores were analysed using univariate or multivariable regression analyses; all analyses were performed using either OpenEpi [10] or SAS Version 9.1.2 [11]. All p-values presented are two-tailed.

## Results

Seventy-seven completed country questionnaires were returned, 21 from G-countries and 56 from NG-countries, a response rate of 80%. Of the 77 responding countries, 45% were OECD low-income countries, 39% low-middle-income countries and 15% upper-middle-income countries.

All countries reported that they had developed a national strategic HIV plan; all G-countries and 54 (95%) NG-countries reported that they had established a national AIDS coordinating authority. Eighteen (86%) G-countries and 44 (77%) NG-countries reported that they had established a national HIV monitoring and evaluation (M&E) system. In terms of the type of data collected through these M&E systems, of the 63 countries that completed this question, all reported that they collected health sector data, 91% collected data on social services, 88% collected geographical information, 86% educational information, 64% economic data and 61% labour data. No significant differences were observed between responses from G- and NG-countries.

### Aggregated analyses

When comparing G- and NG-countries in terms of the aggregate categories, statistically significant higher scores were observed for G-countries compared with NG-countries for *information governance *(p < 0.01) (Table 1). Conversely, for *country policies*, *data collection*, *storage*, *access *and *transfer *categories, NG-countries scores were statistically significantly higher compared with G-countries (Table 1).

**Table 1:** Median (interquartile) and range of aggregate scores for aggregate categories for those countries that reported having developed guidelines (G-countries) and countries intending to develop guidelines in the future (NG-countries)

		Median score (IQR) [range] N = 78			
**Aggregate categories**	**Sub-categories included in each aggregate category**	**Standardized composite score**	***Countries***		

		**Overall**	**G-countries** **N = 21**	**NG-countries** **N = 57**	**p-value** **(Mann-Whitney U test)**

**Information governance**	Privacy law Consent for data collection HIV policy framework M&E framework categories*	69.6 (52.5 to 91.7) [10 to 100]	82.5 (65.0 to 95.8) [52.5 to 100]	65.0 (44.2 to 86.7) [10 to 100]	0.010

**Country policies**	Existence of C&S policy** Development process Policy dissemination Sectoral coverage of policy Existence of site manager for policy Breach management Aspect coverage of policy Governance	80 (72.5 to 100) [0 to 100]	75.7 (63.7 to 92.1) [20 to 100]	83.0 (75.6 to 100.0) [0 to 100]	0.027

**Data collection**	Collection: types Collection: method	59.6 (41.8 to 69.7) [0 to 93.8]	49.5 (26.4 to 59.6) [0 to 87.5]	61.1 (47.1 to 71.2) [0 to 93.7]	0.028

**Data storage**	Storage System availability	69.8 (44.5 to 80.5) [0 to 97.7]	13.6 (0.0 to 60.7) [0 to 73.4]	73.5 (64.3 to 82.8) [0.0 to 97.7]	<0.001

**Data access**	Access: data preparation for dissemination Access: staff preparation Access: internal users Access: external users	65.9 (33.6 to 75.5) [2.5 to 89.1]	26.6 (17.0 to 49.7) [2.5 to 74.7]	71.3 (59.2 to 78.1) [2.5 to 89.1]	<0.001

**Data transfer**	Data transfer	64.3 (21.4 to 71.4) [0 to 100]	0 (0 to 28.6) [0 to 78.6]	64.3 (50.0 to 78.6) [0 to 100]	<0.001

*M&E = Monitoring and evaluation; **C&S = Confidentiality & security

No statistically significant associations were observed between country score and HIV prevalence or per capita GNI (Table 2). Similarly, no statistically significant differences were observed in terms of countries scores for the various OECD countries (Table 3), nor between countries that had received PEPFAR funding and those that did not (Table 4).

**Table 2:** Correlation between standardized country scores for information governance, country policies, data collection, storage, access and transfer categories and country HIV prevalence and gross national income per capita

		HIV prevalence		GNI per capita (Income)	
**Aggregate categories**	**Sub-categories included in each aggregate category**	**Correlation coefficient**	***P value***	**Correlation coefficient**	***P value***

**Information** **governance**	Privacy law Consent for data collection HIV policy framework M&E framework categories*	R = 0.06	*P = 0.600*	0.09	*P = 0.415*

**Country policies**	Existence of C&S policy** Development process Policy dissemination Sectoral coverage of policy Existence of site manager for policy Breach management Aspect coverage of policy Governance	R = 0.02	*P = 0.864*	-0.01	*P = 0.954*

**Data collection**	Collection: types Collection: method	R = 0.06	*P = 0.611*	-0.03	*P = 0.826*

Data storage	Storage System availability	R = 0.11	*P = 0.346*	-0.13	*P = 0.280*

Data access	Access: data preparation for dissemination Access: staff preparation Access: internal users Access: external users	R = 0.06	*P = 0.579*	-0.03	*P = 0.773*

Data transfer	Data transfer	R = 0.15	*P = 0.219*	-0.09	*P = 0.458*

*M&E = Monitoring and evaluation; **C&S = Confidentiality & security

**Table 3:** Comparison of standardized country scores for information governance, country policies, data collection, storage, access and transfer categories and between countries based on OECD income categorization

		Median score (IQR) [range] N = 78					
**Aggregate categories**	**Sub-categories included in each aggregate category**	***Standardized composite score***	***OECD income categorization***				

		**overall**	**High** **N = 1**	**Low** **N = 35**	**Low Middle** **N = 30**	**Upper Middle** **N = 12**	***p-value*** (**Kruskal-Wallis test)**

**Information governance**	Privacy law Consent for data collection HIV policy framework M&E framework categories*	69.6 (52.5 to 91.7) [10 to 100]	56.7	70.0 (54.2 to 95.0) [14.2 to 100.0]	65.4 (52.5 to 90.8) [10.0 to 100.0]	74.6 (44.2 to 95.8) [15.0 to 100.0]	0.889

**Country policies**	Existence of C&S policy** Development process Policy dissemination Sectoral coverage of policy Existence of site manager for policy Breach management Aspect coverage of policy Governance	80 (72.5 to 100) [0 to 100]	100.0	92.7 (73.3 to 100.0) [0 to 100]	80.0 (63.8 to 94.3) [0 to 100]	94.0 (68.6 to 94.0) [20 to 100]	0.111

**Data collection**	Collection: types Collection: method	59.6 (41.8 to 69.7) [0 to 93.8]	62.0	59.6 (43.3 to 73.6) [0 to 87.5]	57.0 (26.4 to 69.7) [0 to 93.8]	56.5 (46.4 to 65.4) [7.7 to 87.5]	0.714

**Data storage**	Storage System availability	69.8 (44.5 to 80.5) [0 to 97.7]	83.8	70.1 (58.4 to 79.2) [0 to 97.7]	64.3 (6.8 to 76.9) [0 to 95.5]	70.9 (60.2 to 82.1) [0 to 90.9]	0.195

**Data access**	Access: data preparation for dissemination Access: staff preparation Access: internal users Access: external users	65.9 (33.6 to 75.5) [2.5 to 89.1]	80.3	66.1 (47.3 to 74.7) [2.5 to 88.9]	53.4 (22.4 to 74.8) [2.5 to 89.1)	71.0 (52.4 to 76.7) [2.5 to 83.5)	0.351

**Data transfer**	Data transfer	64.3 (21.4 to 71.4) [0 to 100]	85.7	64.3 (42.9 to 71.4) [0 to 85.7]	42.9 (0 to 78.6) [0 to 100]	50.0 (32.1 to 78.6) [0 to 85.7]	0.355

*M&E = Monitoring and evaluation; **C&S = Confidentiality & security

**Table 4:** Comparison of standardized country scores for information governance, country policies, data collection, storage, access and transfer categories and between PEPFAR focus, PEPFAR non-focus and non-PEPFAR countries

		Median score (IQR) [range] N = 78			
**Aggregate categories**	**Sub-categories included in each aggregate category**	**PEPFAR**			

		**Non-PEPFAR countries** **N = 56**	**PEPFAR** **non-focus countries** **N = 9**	**PEPFAR** **focus countries** **N = 13**	**P value** (**Kruskal-Wallis test)**

**Information governance**	Privacy law Consent for data collection HIV policy framework M&E framework categories*	69.5 (44.6 to 93.3) [10.0 to 100.0]	65.0 (56.7 to 82.5) [48.3 to 95.0]	70.0 (62.5 to 86.7) [14.2 to 100.0]	0.864

**Country policies**	Existence of C&S policy** Development process Policy dissemination Sectoral coverage of policy Existence of site manager for policy Breach management Aspect coverage of policy Governance	81.6 (75.1 to 98.5) [0.0 to 100.0]	80.0 (65.7 to 96.0) [20.0 to 100.0]	73.3 (61.3 to 100.0) [0.0 to 100.0]	0.623

**Data collection**	Collection: types Collection: method	61.1 (44.5 to 69.7) [0.0 to 93.8]	54.3 (27.9 to 59.6) [0.0 to 87.5]	54.3 (27.9 to 59.6) [0.0 to 87.5]	0.358

**Data storage**	Storage System availability	69.5 (52.9 to 79.5) [0.0 to 97.7]	67.5 (0.0 to 80.5) [0.0 to 87.3]	70.3 (37.2 to 76.5) [0.0 to 88.6]	0.750

**Data access**	Access: data preparation for dissemination Access: staff preparation Access: internal users Access: external users	66.6 (35.6 to 75.9) [2.5 to 89.1]	49.7 (17.5 to 72.0) [2.5 to 83.9]	65.6 (18.5 to 74.8) [2.5 to 83.5]	0.763

**Data transfer**	Data transfer	64.3 (28.6 to 78.6) [0.0 to 100.0]	71.4 (0.0 to 71.4) [0.0 to 78.6)	57.1 (10.7 to 67.9) [0.0 to 78.6]	0.649

*M&E = Monitoring and evaluation; **C&S = Confidentiality & security

### Existing country guidelines

G-countries were asked to provide copies of their relevant policy documents: 13 (62%) countries did so. However, none of these documents provided the degree of detailed guidance described in the Interim Guidelines [3]. Most of the existing guidelines required the strict maintenance of medical confidentiality surrounding HIV-related information, indicating that consent was frequently required for testing and sometimes for sharing HIV information with other professionals or individuals.

Confidentiality exemptions were most commonly made for the purposes of statutory monitoring and reporting, medical referrals and to inform sexual partners. Of the 57 NG-countries, 33 (59%) indicated that they intended to develop their own guidelines, and 10 (29%) reported that they had already started this process.

### Privacy laws

Eighteen (90%) G-countries indicated that they had existing privacy laws, compared with 31 (57%) NG-countries [p < 0.02]. Concerning the requirement to obtain consent for collecting individual data for routine government analyses, 17 (81%) G-countries indicated that such consent was required compared with 30 (54%) NG-countries [p = 0.05]. When asked about collecting data for research purposes, 19 (91%) G-countries and 45 (82%) NG-countries indicated that individual consent was required for the collection of research data, a difference that was not statistically significant.

### Selected confidentiality and security issues

Guidelines on data collection, storage, access, transfer, analysis, use and feedback, which G-countries had included in their policy documents, were similar to those that the NG-countries mentioned that they would include. However, 12 (75%) G-countries reported that data backup and data recovery were covered in their guidelines, while 51 (98%) NG-countries indicated that they would include such topics [p < 0.02]. For data disposal, 11 (65%) G-countries had this covered in their guidelines compared with 49 (96%) NG-countries [p < 0.005] that indicated that they would include this topic.

Seventeen (89%) G-countries and 52 (100%) NG-countries reported that confidentiality needed to be maintained when collecting individual information in a clinical setting. Three G-countries and eight NG-countries reported that this could be done in a secluded area of an open room, while 13 (68%) G-countries reported that this should be done in a closed room compared with 49 (93%) NG-countries [p < 0.05]

Ten (56%) G-countries and 50 (96%) NG-countries [p < 0.0005] reported that the installation of anti-virus software on all computers is, or should be, part of the guidelines. Countries that recognized the need for anti-virus software also identified the need to regularly update such programmes.

Three (17%) G-countries had reported that they had created steering groups to oversee implementation of guidelines on data collection, storage, analysis, use and release, while 42 (81%) NG-countries [p < 0.00005] indicated that they would do so in future. Such an oversight role was considered to be appropriate for health or community facilities, sub-national or national government facilities and national data warehouses.

Concerning the removal of personal identifiers before transferring information to data repositories or warehouses for monitoring and evaluation of services, 11 (65%) G-countries had incorporated this in their policies compared with 51 (98%) NG-countries [p < 0.002] that claimed that they would include them in future guidelines. Provisions for accessing stored information by academics or other stakeholders for specific analyses or projects was reportedly encouraged by 13 (72%) G-countries, while 49 (94%) NG-countries [p < 0.05] indicated that they would include provisions for such access in future guidelines.

## Discussion

Few countries scaling up HIV services had developed guidelines on protecting the confidentiality and security of HIV information at the time of completing the survey. The Interim Guidelines cover three aspects: privacy, confidentiality and security. Based on the feedback from country informants, the relationship between these three is not always understood. For example, 49 countries claimed to have developed privacy laws, including 55% of countries that claimed not to have developed guidelines. It is notable that in the countries that reportedly had developed policy guidelines, their implementation lacked the breadth and depth of those set out in the published UNAIDS/PEPFAR Interim Guidelines [3].

The only area in which the G-countries scored higher than the NG-countries was the *information governance *category, which included the more likely existence of privacy laws in G-countries compared with NG-countries. However, countries may face practical challenges that hinder adoption of certain aspects of the guidelines, while the generally higher scores for NG-countries may also reflect the fact that NG-countries have so far not fully appreciated the level of effort needed to develop comprehensive measures to protect the confidentiality and security of personal HIV information.

The majority of countries without guidelines reported that they wanted to develop such guidelines, while some claimed to have started this process. However, respondents who completed the questionnaire for NG-countries may have treated the questionnaire as a "wish-list", and not all areas described in the Interim Guidelines may be included once countries have developed their own guidelines. This suggests that the implementation of different aspects of such guidelines may pose practical difficulties in resource-limited countries, and that donor countries or international agencies must assist in the adaptation, adoption and implementation in these countries.

For example, the finding that G-countries had included virus protection and data backup procedures into their local policies less frequently than NG-countries intended to do in future policies could be due to the fact that countries lack the resources to maintain such procedures. It could also be due to an under appreciation of the important role that simple and affordable data protections procedures can play in assuring the consistent availability of electronic information systems.

Similarly, no associations were observed between country scores and HIV prevalence, GNI per capita and OECD country ranking. While one could argue that the better-off countries have more resources to implement such measures, this association did not hold. The extreme example of this is that even many industrialized countries have not developed such guidelines, although privacy laws may be more developed in a number of these countries. That countries like the US and UK have recently stepped up activities in this areas may well be related to the fact that they have had numerous examples where personal information has found its way into the hands of unauthorized third parties [12-14].

While the response rate for this survey was high, a number of limitations exist. UNAIDS and PEPFAR staff were asked to get the relevant host country professionals to complete the questionnaires, which may not have been possible in all countries. Even if country professionals completed the questionnaire, not all of them may have been cognisant of all existing legal or other relevant policies in their country. Furthermore, given that the survey dealt with a range of diverse but related issues, some informants may have developed "answering fatigue".

The issue of maintaining the confidentiality and security of HIV information is very important but remains a neglected policy area in many countries. In a number of countries, the fact that government officials were asked to complete the questionnaire facilitated raising the issue of confidentiality and security of HIV information for the first time, and catalyzed stakeholders into action.

## Conclusions

Given the persistence of stigma and discrimination in many countries towards HIV infection and people living with HIV (PLHIV) [15], confidentiality concerns may deter people from being tested for HIV or using services available to PLHIV or people affected by HIV [16,17]. If governments and health professionals can ensure the confidentiality and security of HIV information collected in community or health facilities and information repositories like national data warehouses, more people may come forward to be tested or use services created to serve PLHIV or people affected by HIV [18]. On the whole, PLHIV and people affected by HIV appreciate the need for the availability of accurate and contemporary information to improve clinical management and to monitor and evaluate services, as indicated by the strong involvement that PLHIV had in developing the Interim Guidelines and subsequent responses [18].

One step towards achieving confidentiality and security of HIV information is for more countries to appreciate the breath and depth required to ensure the protection of personal HIV information and to adapt, adopt and implement local guidelines on protecting the confidentiality and security of HIV information in line with their cultural and socio-economic contexts, using the published Interim Guidelines [3] to guide their efforts and having the resources to do so.

## Competing interests

The authors declare that they have no competing interests.

## Authors' contributions

EJB and XS conceived the study, were involved with the design, data collection, analyses and writing the paper. GH was involved with the design of the study, data collection, analyses and writing the paper. SM was involved with the design of the study, analyses and writing the paper. DM and PDL were involved with data interpretation and writing the paper. All authors read and approved the final manuscript.

## References

[B1] United Nations 60/262. Political Declaration on HIV/AIDS 2006 UN New York http://data.unaids.org/pub/Report/2006/20060615_HLM_PoliticalDeclaration_ARES60262_en.pdf (Accessed November 2010)

[B2] 55/2. United Nations Millennium Declaration 2000 United Nations, New York US http://www.un.org/millennium/declaration/ares552e.htm (Accessed November 2010)

[B3] UNAIDS/PEPFAR Interim Guidelines on Protecting the Confidentiality and Security of HIV Information: Proceedings from a Workshop, 15-17 May 2006 2007 Geneva, Switzerland http://data.unaids.org/pub/manual/2007/confidentiality_security_interim_guidelines_15may2007_en.pdf (Accessed November 2010)

[B4] Beck EJ Santas X DeLay P Why and How to Monitor the Cost and Evaluate the Cost effectiveness of HIV Services in Countries AIDS 2008 14 Suppl 1 S75 S85 10.1097/01.aids.0000327626.77597.fa 18664958

[B5] WHO Health Metrics Network Biennial Report 2005/2006 2007 Geneva: WHO http://www.who.int/healthmetrics/governance/HMN_biennial_report.pdf (Accessed November 2010)

[B6] UNAIDS The "Three Ones" in action: where we are and where we go from here 2005 Geneva http://data.unaids.org/publications/irc-pub06/jc935-3onesinaction_en.pdf (Accessed November 2010)

[B7] UNAIDS 2008 Report on the global AIDS epidemic 2008 Geneva

[B8] World Bank Indicators 2008 Washington http://data.worldbank.org/indicator (Accessed November 2010)

[B9] World Bank World Bank Country Classification 2008 Washington http://www.oecd.org/dataoecd/15/29/35876528.pdf (Accessed November 2010)

[B10] Open Source Epidemiologic Statistics for Public Health: OpenEpi version 2.3 http://www.openepi.com (Accessed November 2010)

[B11] SAS Version 9.1.2 SAS Institute Inc., Cary, NC, USA

[B12] BBC UK's families put on fraud alert 2007 http://news.bbc.co.uk/2/hi/7103566.stm (Accessed November 2010)

[B13] Hamilton BA Medical Identity Theft Environmental Scan 2008 Rockville, MD, USA http://www.hhs.gov/healthit/documents/IDTheftEnvScan.pdf (Accessed November 2010)

[B14] Nagesh G HHS adopts new rules to coordinate health care technology NextGov 2009 http://www.nextgov.com/nextgov/ng_20090122_2204.php (Accessed November 2010)

[B15] Mahajan Anish P Sayles Jennifer N Patel Vishal A Remien Robert H Sawires Sharif R Ortiz Daniel J Szekeres Greg Coates Thomas J Stigma in the HIV/AIDS epidemic: a review of the literature and recommendations for the way forward AIDS 2008 14 S67 S79 18641472 10.1097/01.aids.0000327438.13291.62 PMC2835402

[B16] Rahmati-Najarkolaei F Niknami S Aminshokravi F Bazargan M Ahmadi F Hadjizadeh E Tavafian SS Experiences of stigma in healthcare settings among adults living with HIV in the Islamic Republic of Iran Journal of the International AIDS Society 2010 14 27 http://www.jiasociety.org/content/13/1/27 (Accessed November 2010) 10.1186/1758-2652-13-27 20649967 PMC2919446

[B17] Duff P Kipp W Wild TC Rubaale T Okech-Ojony J Barriers to accessing highly active antiretroviral therapy by HIV-positive women attending an antenatal clinic in a regional hospital in western Uganda Journal of the International AIDS Society 2010 14 37 http://www.jiasociety.org/content/13/1/37 (Accessed November 2010) 10.1186/1758-2652-13-37 20863399 PMC2954932

[B18] Hargreaves S New guidance issued on HIV data collection The Lancet Infectious Diseases 2007 14 508 10.1016/s1473-3099(07)70171-8 17695649

